# Degradation of QPPO-based anion polymer electrolyte membrane at neutral pH

**DOI:** 10.1039/d3ra02889e

**Published:** 2023-07-05

**Authors:** Zhiming Feng, Gaurav Gupta, Mohamed Mamlouk

**Affiliations:** a School of Engineering, Newcastle University Merz Court Newcastle upon Tyne NE1 7RU UK z.feng12@newcastle.ac.uk; b Chemical Engineering, Lancaster University Lancaster LA1 4YW UK; c School of Engineering, Newcastle University Merz Court Newcastle upon Tyne NE1 7RU UK

## Abstract

The chemical stability of anion polymer electrolyte membranes (AEMs) determines the durability of an AEM water electrolyzer (AEMWE). The alkaline stability of AEMs has been widely investigated in the literature. However, the degradation of AEM at neutral pH closer to the practical AEMWE operating condition is neglected, and the degradation mechanism remains unclear. This paper investigated the stability of quaternized poly(*p*-phenylene oxide) (QPPO)-based AEMs under different conditions, including Fenton solution, H_2_O_2_ solution and DI water. The pristine PPO and chloromethylated PPO (ClPPO) showed good chemical stability in the Fenton solution, and only limited weight loss was observed, 2.8% and 1.6%, respectively. QPPO suffered a high mass loss (29%). Besides, QPPO with higher IEC showed a higher mass loss. QPPO-1 (1.7 mmol g^−1^) lost nearly twice as much mass as QPPO-2 (1.3 mmol g^−1^). A strong correlation between the degradation rate of IEC and H_2_O_2_ concentration was obtained, which implied that the reaction order is above 1. A long-term oxidative stability test at pH neutral condition was also conducted by immersing the membrane in DI at 60 °C water for 10 months. The membrane breaks into pieces after the degradation test. The possible degradation mechanism is that oxygen or OH˙ radicals attack the methyl group on the rearranged ylide, forming aldehyde or carboxyl attached to the CH_2_ group.

## Abstract 2

The degradation of QPPO-based anion exchange membrane in DI water.

## Graphical abstract

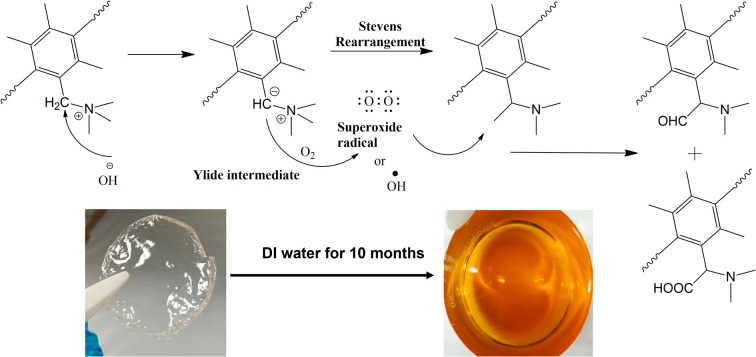

## 1 Introduction

The anion polymer electrolyte membrane (AEM) is one of the significant components in AEM water electrolyzers (AEMWE).^1^ It acts as the ion conductor and the barrier between the anode and cathode catalyst layers.^2–6^ There are several requirements for an AEM to ensure the durable operation of the system, including high hydroxide (OH^−^) conductivity, good mechanical properties, and superior chemical stability to ensure the highly efficient and long-term operation of the system.^7–9^ The conductivity has substantially increased over the last decade.^9–11^ However, chemical stability is still a formidable challenge for AEM, significantly affecting the electrolyzer's durability.^12^ The attack of ions and radicals on the backbone and functional group leads to the chain scission and loss of functional groups.^13–15^ The membrane will become thinner and eventually decompose, failing to function as a gas and electrical separator. Yoo and co-workers studied the chemical stability of different types of anion exchange membranes, including poly(arylene ether), poly(ether imide) and poly(arylene ether).^16–19^ They prepared the composite membrane to improve the chemical stability.^19^ For example, the poly(phenylene oxide) based membrane was enhanced by using graphitic carbon nitride (gC_3_N_4_) derivatives (porous (p-) gC_3_N_4_) and F-doped porous ((F-p-) gC_3_N_4_). The membrane assembly electrode demonstrated operated at 60 °C for 100 h under 0.15 A cm^−2^ current density.^19^

The chemical resistance of AEMs in the literature mainly focused on alkaline stability tests, for example, in N_2_ saturated high temperature/high alkaline environment. The degradation mechanism of AEM under the alkaline environment was investigated clearly. The membrane suffered from Hoffman elimination, nucleophilic substitution, and formation of ylide intermediates.^20,21^ However, the practical working condition of AEMs is oxygen (and hydrogen) rich, nearly neutral-pH condition if deionized water is fed.^22^ Limited research was conducted to investigate the degradation mechanism of AEM under neutral-pH conditions. Besides, the testing conditions for oxidative stability were not standardized in the literature. Several ways exist to evaluate polymers' oxidative stability.^23–26^ Typically, oxidative stability can be assessed in the air or oxygen-saturated DI-water, H_2_O_2_, or Fenton's solution, as is shown in Table 1. The primary methodology is to immerse the membrane in the oxidative media for a certain period. The characteristic parameters are measured, such as the membranes molecular weight, tensile strength, weight, conductivity, IEC losses, *etc.*

**Table 1:** The properties of different membranes in Fenton solution at 60 °C for 25 h

Membrane	Initial weight (mg)	Final weight (mg)	Weight loss (%)
FAA-3-30 (1.45 mmol g^−1^)^a^	110.3	91.5	17.0
FAA^a^	238.1	217.1	8.8
SEBS^b^	246.3	245.5	0.3
PPO	252.3	245.3	2.8
ClPPO (this work)	191.4	188.3	1.6
LDPE^b^	172.9	177.9	0.73
QPPO^c^ (1.3 mmol g^−1^, this work)	151.6	106.4	29.2

a The commercial membranes.

b The pristine membrane.

c The membrane prepared by the pristine PPO polymer.

Both HO˙ and 
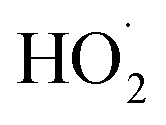
 are reactive oxygen species (ROS) that react with polymers and contribute to their degradation.^27,28^ The Fenton reaction is an effective oxidant of various organic substrates and can produce oxygen radicals.

**Fig. 1:**
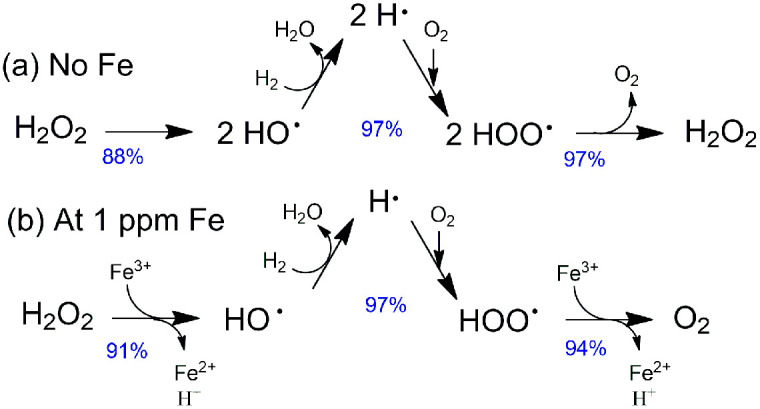
The main reaction pathways start from H_2_O_2_ with (a) and without (b) Fe. Reproduced with permission from ref. 13. Copyright (2011), The electrochemical society.

Fenton's solution (3 wt% H_2_O_2_ with 2 ppm Fe^2+^) is prepared by adding Fe^2+^ into the H_2_O_2_ solution. The primary processes for radical generation are shown in Fig. 1.^13^ However, the lifetime, reactivity, and generation reaction rates of radicals are temperature-dependent. Unfortunately, even when using Fenton reagents for AEM oxidative stability study, these were done for different durations and temperatures, making comparisons difficult.

Other strategies were adopted to evaluate oxidative stability, *e.g.*, a visual approach to membrane integrity. Zhu and co-workers synthesized highly conductive and alkaline-stable AEMs-based on midblock-quaternized triblock copolystyrenes.^29^ They evaluated the oxidation stability by immersing the membranes in the Fenton solution and observing changes in their physical properties, such as the color and shape, that indicated degradation. Liu and co-workers prepared a series of AEMs-based on chloromethylated polysulfone incorporated with quaternized graphenes.^30^ The pristine membranes were soaked into a hot Fenton solution at 80 °C under stirring to examine the oxidative stability. The Fenton reagent was refreshed every 4 hours. The deformation of the membranes was observed, such as changes in colour and physical shape. Xiong and co-workers synthesized quaternized cardo polyetherketone AEM.^31^ The oxidative stability was measured by measuring the weight loss at given time intervals after immersing the samples in a 3 wt% H_2_O_2_ solution at 60 °C. After 168 h, the weight loss of the pristine and quaternized membranes was 3% and 2.1%, respectively. Maurya and co-workers prepared vinylbenzyl chloride (VBC)-based AEM crosslinked by divinylbenzene (DVB) using polyethylene (PE) substrate with different functional groups for vanadium redox flow battery.^24^ They incorporated different functional groups with the same anion exchange composite membranes, such as ammonium, diammonium, and phosphonium. Espiritu *et al.* studied the oxidative stability of LDPE-based AEMs with vinylbenzyl chloride (VBC) grafts. They found the loss of the functional and VBC groups.^32^

Poly(*p*-phenylene oxide) (PPO) based membranes were widely investigated due to their excellent physicochemical properties, such as high transition temperature (*T*_g_), excellent mechanical strength and good chemical stability.^33^ The degradation mechanism of the QPPO-based AEM with trimethylamine (TMA) head group is explained clearly in alkaline media, but not in neutral pH media, which is more relevant to the practical operating condition of water electrolyzer with fed water. The quaternized PPO was prepared and characterized in our previous study.^34^ In this paper, the degradation mechanism of PPO-based membranes at neutral pH condition was studied and discussed. The changes in weight and IEC were recorded. The membrane stability was tested in different solutions, including H_2_O_2_ solution, Fenton solution, and DI water. The insights gained provide a better understanding of the degradation process of quaternary ammonium-functionalized membranes in a neutral environment and guidelines for structural design and modification of AEMs to improve the stability against radical-induced ageing.

## 2 Experimental

### 2.1 Materials

Poly(2,6-dimethyl-1,4-phenylene oxide) (PPO), *N*-methyl-2-pyrrolidinone (NMP), 1,3,5-trioxane, chlorotrimethylsilane (TMCS), SnCl_4_, chloroform, trimethylamine (TMA, 45 wt% in H_2_O), potassium hydroxide (KOH), ferrous sulfate (FeSO_4_), methanol, sodium chloride, and hydrogen were purchased from Sigma-Aldrich and used without further purification.

### 2.2 Preparation of PPO-based anion polymer electrolyte membrane

**Fig. 2:**
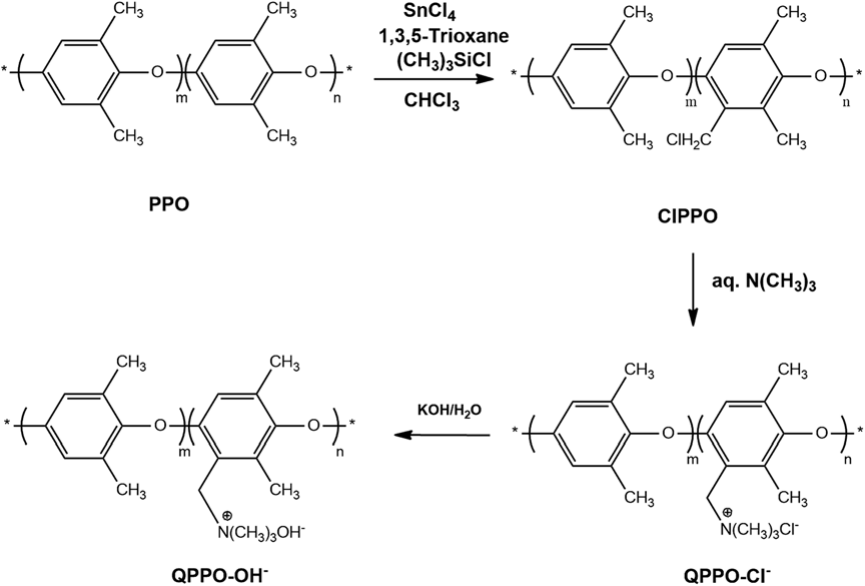
Synthetic route for QPPO-based AEM.^34^

The AEMs were synthesized by the Friedel–Crafts reaction of PPO, 1,3,5-trioxane and chlorotrimethylsilane under SnCl_4_ catalyst, as previously reported *via* the sequential steps, including chloromethylation, quaternization and ion exchange.^34^Fig. 2 shows the synthetic routes for QPPO-based AEM functionalized with TMA.

### 2.3 Ion exchange capacity

Ion exchange capacity (IEC) was calculated by using acid–base titration with Methyl red as the indicator. Before titration, the membrane in hydroxide form was immersed in a known volume of 1 M NaCl solution for 24 h to liberate the hydroxide ions. Then, 10 ml of the solution was titrated with a known concentration of sulfuric acid (H_2_SO_4_) solution until colour change was observed. Then, the membrane was dried in the oven overnight at 60 °C and weighed. The IEC was calculated using the amount of OH^−^ in millimoles divided by dry membrane weight in grams.

### 2.4 Structure characterization

FTIR and NMR were used to characterize the structure of the polymer. A Varian 800 FT-IR in Attenuated Total Reflectance (ATR) mode was used to verify the successful introduction of functional groups. ^1^H NMR and ^13^C NMR spectra were recorded on a Bruker Av-400-WB instrument using CDCl_3_ as solvent.^11,35^ Besides, HSQC (Heteronuclear Single Quantum Coherence) NMR technique was also characterized.

### 2.5 Oxidative stability

The membranes were immersed in N_2_-saturated DI water at room temperature and 60 °C for 10 months. The samples were sealed in plastic test bottles to minimize exposure to atmospheric CO_2_, which can react with OH-counterion to produce carbonate/bicarbonate.

The weight and IEC were measured before and after tests. Besides, the membrane was immersed in Fenton's solution (3 wt% H_2_O_2_ added 2 ppm FeSO_4_) at 60 °C for 24 h. The IEC and weight loss change were calculated on the data before and after treatment.

## 3 Results and discussion

### 3.1 Oxidation stability of AEM backbone

Several in-house and commercial polymer membranes and their functionalized chloromethylated and quaternized (headgroup) were immersed in a Fenton solution at 60 °C for 25 h. The loss of weight is shown in Table 1.

The pristine polymers (backbone), including polystyrene-*b*-poly (ethylene/butylene)-*b*-polystyrene (SEBS), PPO, chloromethylated PPO (ClPPO) and low-density polyethylene (LDPE), showed very slight weight loss after 25 h. This means they exhibit good oxidative stability. It is worth mentioning. However, that pristine PPO had slightly higher weight loss than LDPE and SEBS. Interestingly, adding the functional chloromethylated group to PPO didn't decrease its oxidation stability but improved it, possibly due to the addition of chlorine. Chlorine's electron-withdrawing nature can reduce the susceptibility of the membrane to oxidation, preventing the degradation of the polymer backbone under oxidative conditions.^36^ However, after quaternization (head group addition), FAA-3-30 (1.5 mmol g^−1^) and QPPO (1.3 mmol g^−1^) showed significant weight loss and reduction in their oxidative stability. The weight loss seen cannot be explained by the loss of the head group alone. Notably, the membranes were broken into small pieces suggesting significant damage to the backbone and chain session. The degradation mechanism will be studied further below.

### 3.2 The effect of IEC on the degradation in H_2_O_2_ solution

The degradation of QPPO-based membranes with different IEC was studied by immersing the membranes in 0.5 wt% H_2_O_2_ solutions for 1 h. As shown in Table 2, PPO-based membranes with different IEC of 1.7 and 1.3 mmol g^−1^ at the fixed crosslinking degree of 5%. After the degradation test, QPPO-1 and QPPO-2 lost the same level of IEC of *ca.* 6%. For mass loss, QPPO-1 lost nearly twice as much mass as QPPO-2. If the lost mass is only caused by headgroup loss, then the estimated loss in QPPO-1 should be a factor of 1.3 of that of QPPO-2.

**Table 2:** The comparison of PPO-based membrane in H_2_O_2_ solution with different IEC after degradation test

Sample	H_2_O_2_ conc. (wt%)	*T* (°C)	IEC^a^ (mmol g^−1^)	*t* (h)	IEC loss (%)	Weight loss (%)
QPPO-1	0.5	60	1.7	1	5%	9%
QPPO-2	0.5	60	1.3	1	6%	4%

a Error range: ±0.1.

The significantly higher factor of 2 suggests that backbone loss might also occur. Notably, an increase in IEC promotes oxidative degradation. This could be due to a more significant swelling ratio/water uptake and consequently improved mass transport of ROS in water channels to attack sites or the negative effect of charge distribution on AEM backbone stability from the addition of a positive headgroup or headgroup catalytic/mediator role in the acceleration of ROS attack of vulnerable AEM sites.

### 3.3 The effect of H_2_O_2_ concentration on QPPO AEM degradation

Apart from different IEC, the effect of H_2_O_2_ concentration on the degradation was also investigated. The initial IEC of the PPO-based membrane is 1.6 mmol g^−1^, and the average initial weight was between 110 to 120 mg.

**Fig. 3:**
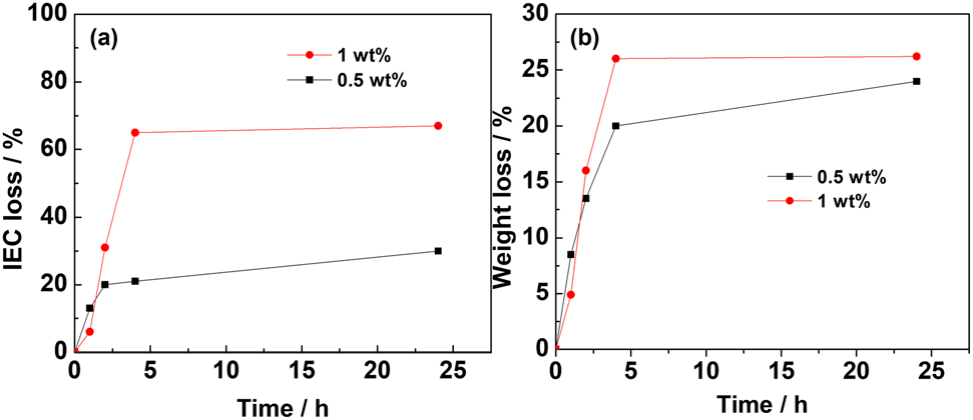
IEC loss (a) and weight loss (b) of QPPO-based membrane in 0.5 wt%, 1 wt% H_2_O_2_ concentration at 60 °C.

Fig. 3(a) illustrates the IEC loss of QPPO-based membrane during degradation in 1 wt% and 0.5 wt% H_2_O_2_ solutions. Given that the studied reaction temperature was 60 °C where the rate of H_2_O_2_ decomposition can be rapid, it can only be assumed that H_2_O_2_ concentration in solution remained constant for short periods below 5 h. The change of H_2_O_2_ concentration due to reaction with AEM polymer can be neglected due to the small mass of membrane sample used. It can be seen from Fig. 3(b) that the rate of mass loss or backbone degradation wasn't affected significantly despite doubling H_2_O_2_ concentration with mass loss of *ca.* 26% after 4 h. The observation above on backbone/mass loss rate increase with an increase in IEC suggests that the headgroup controls the rate-limiting step. In other words, the reaction can be catalyzed by the headgroup or by other polymer locations activated on the headgroup introduction. It could also suggest that there are two parallel degradation mechanisms. One involves oxidation of the head group. The other requires weight loss, not due to oxidation but polymer fragmentation and dissolution, as discussed further below. On the other hand, Fig. 3(a) shows a strong correlation between the degradation rate of IEC and H_2_O_2_ concentration. Reaction order above 1 was seen.

After 4 hours, there were remarkable IEC loss differences by a factor of >3 (20% *vs.* 65%), close to that of second-order reaction when the QPPO-based membrane degraded in 1 wt% H_2_O_2_ than in 0.5 wt% H_2_O_2_. This can be since H_2_O_2_ decomposes into 2 OH radicles which in turn carry out the degradation reaction, possibly attacking two headgroup locations. The rapid loss of head groups of *ca.* 65% after 4 h in 1% H_2_O_2_ shows the vulnerability of benzyl or aryl trimethylammonium to oxidative attacks. Previously, it has been demonstrated that the benzylic carbon linking the tethered head group is a vulnerable point (Ph–CH_2_–TMA). From the results, it can be concluded that the slower degradation of the backbone is most likely caused by the negative effect of charge distribution on AEM backbone stability from adding a positive headgroup.

The larger swelling ratio/water uptake and consequently improved mass transport of ROS in water channels can be excluded as higher H_2_O_2_ concentration didn't increase the mass loss rate. Similarly, the headgroup catalytic/mediator role in accelerating ROS attack of vulnerable AEM sites is unlikely to be the cause. Otherwise, the effect of headgroup loss of *ca.* 65% should have been seen on the rate of mass loss after 4 h unless the effect continues to take effect in solution if headgroup is lost as the whole aryl trimethylammonium as has seen detected in radiation grafted LDPE AEMs.^37^

### 3.4 Comparison between QPPO and LDPE-based membrane

A membrane with excellent oxidation resistance will increase the lifetime of AEMWE. TMA-functionalised vinylbenzyl chloride grafted on low-density polyethylene backbone (LDPE-*g*-VBC-TMA), previous AEMs synthesized by our groups, were used as the benchmark. LDPE-*g*-VBC-TMA showed a high ion conductivity (101 mS cm^−1^ at 60 °C) at high IEC (*ca.* 2.8 mmol g^−1^). At the same time, the OH^−^ conductivity of SEBS was 140 mS cm^−1^ at 50 °C when its IEC was 1.9 mmol g^−1^. As stated above, chemical stability is vital for membranes to guarantee the lifetime of a water electrolyzer, especially oxidative resistance.

**Fig. 4:**
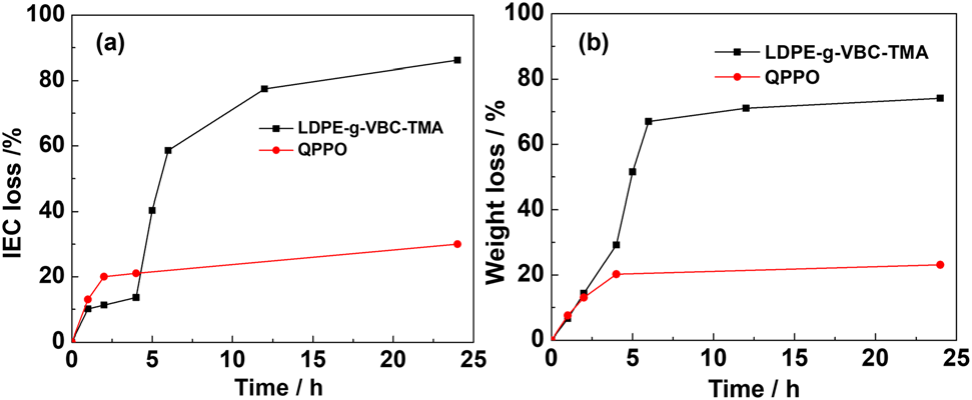
IEC (a) and weight loss (b) of LDPE-*g*-VBC-TMA and QPPO-based membrane in 0.5 wt% H_2_O_2_ at 60 °C.

Fig. 4 shows the IEC and weight loss of LDPE-*g*-VBC-TMA and QPPO in 0.5 wt% H_2_O_2_ for a different time at 60 °C. Initially, the IEC loss of LDPE-*g*-VBC-TMA increased slowly. Then there was a sharp decrease within the first 6 h. After 24 h, the IEC became 0.36 mmol g^−1^ and lost almost 85% of the initial.^32^ However, QPPO showed a slower rate of IEC. It only lost around 20% after 2 h and remained *ca.* 70% after 24 h. This observation suggests that the QPPO structure might be more oxidative stable than VBC-TMA due to a lack of benzylic carbon, resulting in a chain session of the VBC-TMA head group. Another factor contributing to a higher IEC loss rate in LDPE-VBC-TMA AEM is that the membrane is radiation grafted. Hence, depending on graft length, there will be a significant loss of head group per ROS attack compared to QPPO AEM. LDPE-VBC-TMA has higher IEC, which results in higher IEC rate loss, as seen and discussed above.

The weight loss of both studied AEMs had a similar trend as the IEC loss. The weight loss of LDPE-*g*-VBC-TMA was rapid in the first 6 h, reaching 68%. After 24 h, only 25% of the membrane's initial weight remained. The weight loss for PPO was lower than that of LDPE-*g*-VBC-TMA after 24 h. The higher oxidative stability of aromatic QPPO-based AEM can explain this. As discussed above, the stability of the backbone will be altered on functionalization; however, QPPO remains more oxidation resistant than LDPE-VBC-based AEM.

### 3.5 Long-term oxidative stability test in DI water

**Fig. 5:**
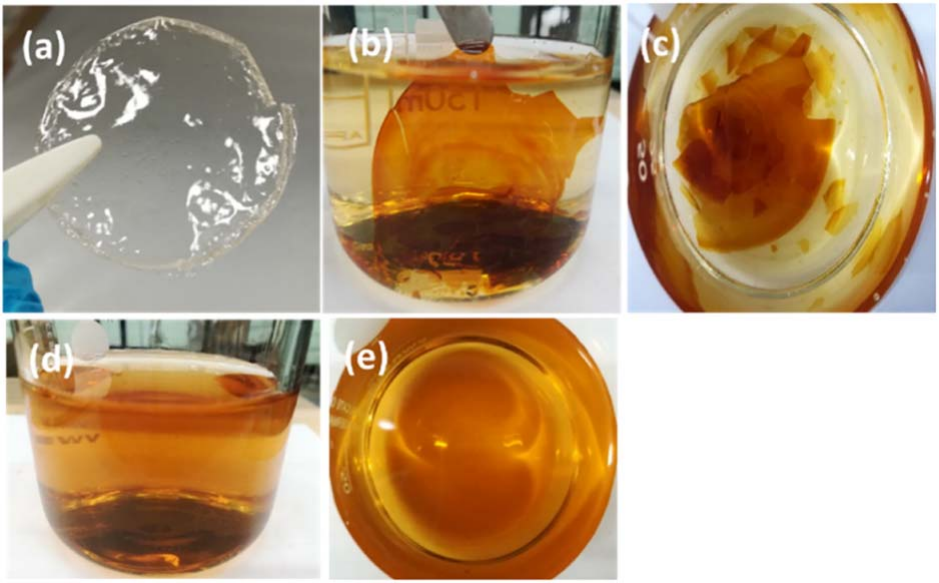
Morphological comparison of original QPPO (a), QPPO after degradation test in 1 M KOH solution (b and c) and DI water (d and e) at 60 °C for 10 months.

To further test the oxidative stability in a neutral environment for the long term, QPPO-14-based membranes were immersed in DI water at 60 °C for 10 months. The morphological changes in the membranes are shown in Fig. 5. Fig. 5(a) shows the original transparent QPPO-based membrane. Fig. 5(b) and (c) reveal the membrane immersed in 1 M KOH solution, which broke into small pieces, while Fig. 5(d) and (e) show the membrane immersed in DI water which became water-soluble and fully dissolved in DI water after 10 months. It is common for ion exchange polymers to lose some mass with time when immersed in water. This is because of the highly hydrophilic nature of the head group. Even commercial perfluorinated polymers with a low IEC of 0.91 mmol g^−1^ (Nafion or SPEEK) undergo weight loss due to water solubility with time, especially at elevated temperatures. As discussed, the solubility can be reduced by reducing IEC and increasing crosslinking. Both samples underwent a colour change (brown) from yellow, suggesting an ageing process.^38^ The result illustrated that the backbone of PPO in DI water suffered a more severe fracture than in just the functional group. It wasn't possible to carry on other tests, for example, change in IEC, as the membranes became too brittle after the degradation test and broke into pieces.

**Fig. 6:**
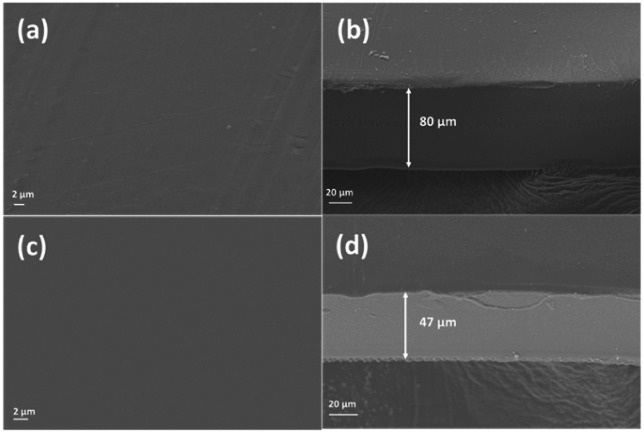
The SEM images of surface (a, c) and cross-section (b, d) of PPO based membrane before and after the degradation test.

The morphology changes were analysed by using scanning electron microscopy (SEM). As is shown in the surface images from Fig. 6(a) and (c), the membranes are dense and flat before and after the degradation test. After the degradation test in the H_2_O_2_ solution, the membrane surface became smoother than the pristine one due to the high swelling. The thickness of the membrane decreased from 80 μm to 47 μm.

To understand the degradation mechanism further, structure characterization was conducted, including FTIR and NMR.

**Fig. 7:**
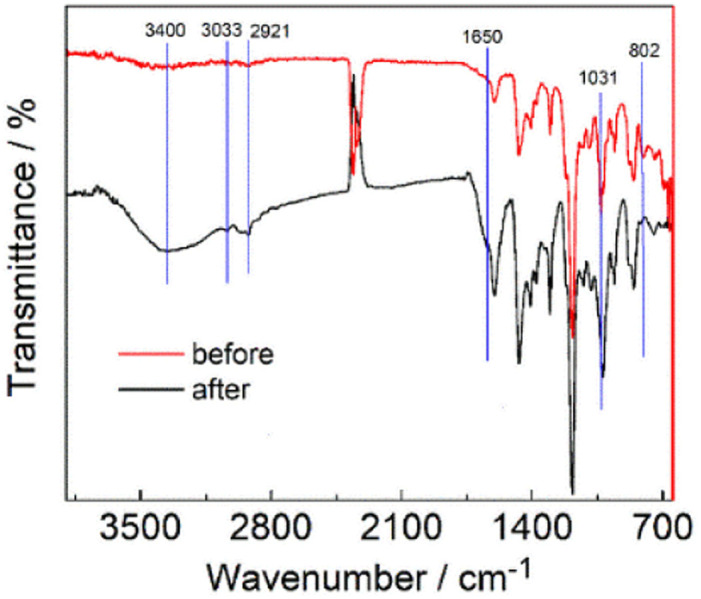
FTIR of the QPPO-based membrane before and after the chemical stability test in DI water for 10 months.

**Fig. 8:**
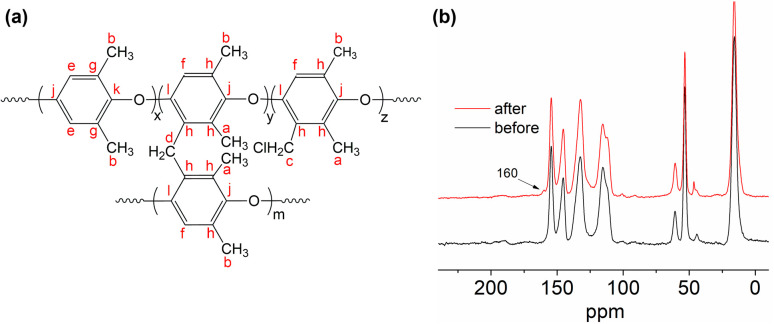
(a) The structure of QPPO and corresponding carbon shift. (b) The solid-state NMR of QPPO-based membrane before and after degradation test.

As is shown in Fig. 7, the QPPO-based membrane before and after the chemical stability test in DI water for 10 months was characterized by FTIR. The peak at 802 cm^−1^ is the C–H bending of benzene ring. The peak at 1031 cm^−1^ is the C–O stretching vibration of the ether group.^39^ The new broad peak round 3102–3423 cm^−1^ is assigned to hydroxyl groups (O–H), and the new peak at 1650 cm^−1^ is the C=O of the carbonyl group.^40,41^ The carboxyl group might be obtained from the oxidation of the PPO-based membrane after the ylide rearrangement. Besides the FTIR, NMR was also adopted to characterize the degradation process. The evidence of CH_2_–C=O can also confirm it in C NMR at 23 ppm, which can be found in the following discussion section. The structure of QPPO is shown in Fig. 8(a). After the degradation test, the degraded sample was characterized by solid-state ^13^C NMR (SSNMR). As is shown in Fig. 8(b), these two spectra almost entirely overlap. One new peak was observed at 160 ppm after degradation, designated to carbonyl (C=O). The small peak around 40 ppm aromatic–CH_2_–Cl is a small amount of the polymer that TMA couldn't reach. It is relatively large, showing a considerable amount of chloromethylated group was not quaternized. This can explain why IEC is lower than theoretical values. There is a new peak at around 45 ppm, which should also be similar to C-aromatic. The small hump peak around 30 ppm corresponds to Ph–CH_2_–Ph of crosslinking.

**Fig. 9:**
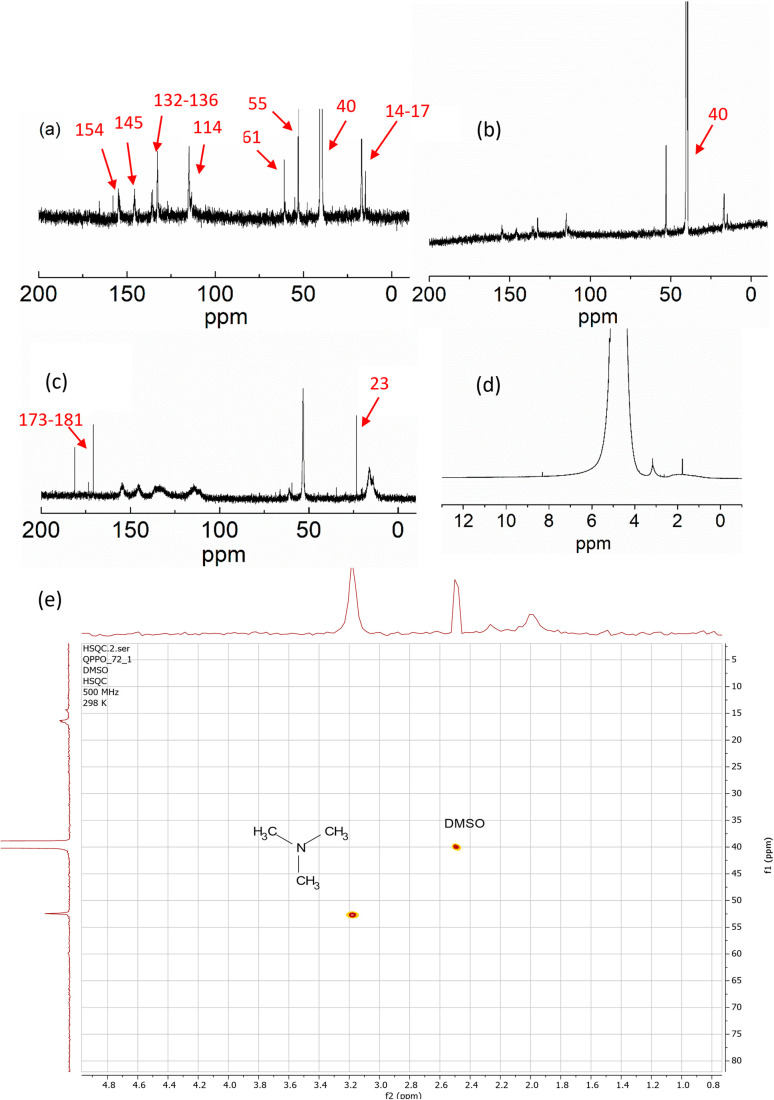
^13^C NMR of (a) QPPO before degradation test, (b) extracted sample after degradation test (c) the residual degradation solution and (d) the ^1^H NMR of the residual degradation solution, (e) HSQC NMR characterization of the residual solution after degradation test in H_2_O_2_ solution.

To further analyze the changes in aged samples, more characterizations were conducted. Fig. 8(a) shows the ^13^C NMR of QPPO dissolved in DMSO-d_6_ before the degradation test, which has almost the same peaks as those seen in ^13^C solid-state NMR (Fig. 8(b)). As is shown in Fig. 9(a), the signal between 114 ppm to 154 ppm corresponds to the aromatic carbon in the benzyl ring. The signal at 61 ppm was assigned to the benzylic carbon of head group C–N.^42^

The signal at 40 ppm is that of Ar–CH_2_–Cl, suggesting despite immersion in TMA solution, not all the chloromethylated group could be reached, reacted and quaternized.

The signal at 53 ppm corresponded to the C–N of methyl groups of TMA. After the degradation test, the remaining broken solids of the membrane were extracted/filtered and dissolved in DMSO-d_6_ and characterized by ^13^C NMR. Fig. 9(b) shows the remaining degraded AEM sample after dissolving in DMSO-d_6._Fig. 9(c) showed ^13^C NMR of the H_2_O solution used for the degradation study containing soluble degradation products, and compared with Fig. 9(a), new peaks around 173 and 181 ppm appear in the dissolved degradation products, corresponding to carboxylic carbon C=O carboxylic acid and aldehyde, respectively. The signal at 23 ppm is due to H_2_C–C=O aliphatic carbon adjacent to the carbonyl carbon.

Interestingly, the signal at 40 ppm corresponding to the chloromethyl group has disappeared, while the quaternary ammonium group at 55 and 61 remained. This suggests that part of the QPPO polymer is water-soluble or becomes water-soluble and dissolved in solution with the headgroup. In addition, there is a soluble oxidation product producing a carboxylic acid group detected. The solid fraction remaining of AEM after the degradation test seems to be the same as that of unaged QPPO with an unreacted chloromethylated group (40 ppm). This suggests that QPPO contains two segments. One has higher quaternization of chloromethylated group and hence highly charged and hydrophilic, resulting in water solubility with time at higher temperatures. The other section contains a higher fraction of unquaternized chloromethyl group, which remained solid. The loss of a soluble segment of QPPO results in membrane breakage in smaller pieces containing the water-insoluble fraction with lower quaternization.

**Fig. 10:**
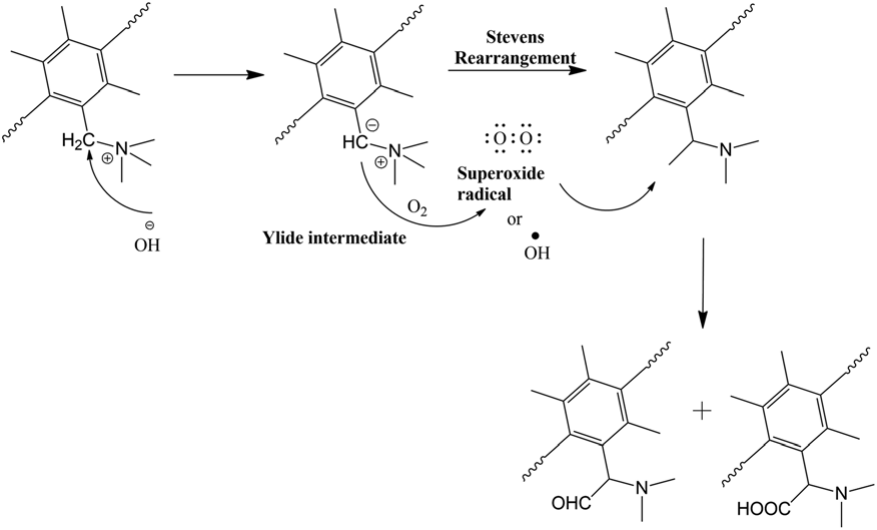
The degradation mechanism of QPPO-based membrane.

It can be concluded that there are two degradation processes involving weight loss due to fragmentation of QPPO where fragments with higher quaternization (amination of introduced chloromethylated groups) and higher hydrophilicity become water-soluble leaving behind fragments with lower quaternization with a higher proportion of un-aminated chloromethylated PPO. The other degradation mechanism involves IEC loss. This involves the established yield formation in addition to OH˙ radicals' formation. As shown in Fig. 10, the ylide formation proceeds for Stevenson rearrangements to produce *N*,*N*-dimethyl-phenylethylamine resulting in seen loss of IEC. The ethylamine group contains vulnerable beta carbon (ethyl group), which can undergo oxidation to form a carbonyl group (aldehyde), which can oxidize further to form a carboxylic group which was observed. The ^1^H NMR of the residual degradation solution was also tested (Fig. 9(e)). The signal around 3.2 ppm is expected to be C–H of amine. The signals were unclear due to the low concentration of the dissolved residue in the solution. To confirm the study, we characterize the residual solution after the degradation test in the H_2_O_2_ solution by using HSQC (Heteronuclear Single Quantum Coherence) NMR technique, in which the correlations between hydrogen (proton) and heteronuclei, such as carbon-13 (^13^C) are measured to obtain valuable information. As is shown in Fig. 9(d), the signal at 3.2 ppm in 1H NMR is related to 53 ppm in 13 NMR, confirming the structure of methyl groups of TMA.

The oxygen or OH˙ radical attacks the methyl group on the rearranged ylide and forms aldehyde or carboxyl attached to the CH_2_ group, explaining the NMR signatures.

## 4 Conclusion

Despite extensive studies on the alkaline stability of AEMs, the degradation mechanism of AEMs in pH-neutral media is neglected. This paper tested the oxidative stability of different membranes in a Fenton solution at 60 °C for 25 h. The membranes based on different backbones were tested in Fenton solution, including FAA-3-50, SEBS, PPO, ClPPO, QPPO and LDPE. The non-quaternized membrane (SEBS, LDPE, PPO and ClPPO) showed good oxidative stability and limited weight loss was observed, 0.3%, 2.8%, 1.6% and 2.8%, respectively. After the functionalization of PPO, the QPPO-based membrane suffered 29% mass loss. The effect of IEC and H_2_O_2_ concentration on QPPO AEM degradation was studied. A strong correlation between the degradation rate of IEC and H_2_O_2_ concentration was obtained, which implied that the reaction order is above 1. More considerable swelling ratio/water uptake and consequently improved mass transport of ROS in water channels to attack sites, or adverse effect of charge distribution on AEM backbone stability from the addition of positive headgroup, or headgroup catalytic/mediator role in the acceleration of ROS attack of vulnerable AEM sites. QPPO-based membrane shows better oxidative stability than LDPE-*g*-VBC-TMA based AEM. The degradation mechanism of PPO-based membrane under DI water conditions was studied. The residual degradation solution and extracted sample after the degradation test were characterized by NMR. The possible degradation mechanism is that oxygen or OH˙ radicals attack the methyl group on the rearranged ylide, forming aldehyde or carboxyl attached to the CH_2_ group.

## Conflicts of interest

There are no conflicts to declare.

## Supplementary Material
